# Unimodal and cross-modal identity judgements using an audio-visual sorting task: Evidence for independent processing of faces and voices

**DOI:** 10.3758/s13421-021-01198-7

**Published:** 2021-07-12

**Authors:** Nadine Lavan, Harriet M. J. Smith, Carolyn McGettigan

**Affiliations:** 1 Department of Speech, Hearing and Phonetic Sciences, University College London, London, UK; 2 Department of Biological and Experimental Psychology, School of Biological and Chemical Sciences Queen Mary University of London, Mile End Road, London, E1 4NS UK; 3 Department of Psychology, Nottingham Trent University, Nottingham, NG1 4FQ UK

**Keywords:** Face, Voice, Unimodal, Cross-modal, Identity perception, Sorting

## Abstract

Unimodal and cross-modal information provided by faces and voices contribute to identity percepts. To examine how these sources of information interact, we devised a novel audio-visual sorting task in which participants were required to group video-only and audio-only clips into two identities. In a series of three experiments, we show that unimodal face and voice sorting were more accurate than cross-modal sorting: While face sorting was consistently most accurate followed by voice sorting, cross-modal sorting was at chancel level or below. In Experiment 1, we compared performance in our novel audio-visual sorting task to a traditional identity matching task, showing that unimodal and cross-modal identity perception were overall moderately more accurate than the traditional identity matching task. In Experiment 2, separating unimodal from cross-modal sorting led to small improvements in accuracy for unimodal sorting, but no change in cross-modal sorting performance. In Experiment 3, we explored the effect of minimal audio-visual training: Participants were shown a clip of the two identities in conversation prior to completing the sorting task. This led to small, nonsignificant improvements in accuracy for unimodal and cross-modal sorting. Our results indicate that unfamiliar face and voice perception operate relatively independently with no evidence of mutual benefit, suggesting that extracting reliable cross-modal identity information is challenging.

## Introduction

Faces and voices provide information about a person’s identity. Current models of person perception argue for various similarities in the way face and voice signals are processed (Belin et al., 2004; Campanella & Belin, 2007; Yovel & Belin, 2013), but there are also notable differences (Young et al., 2020). Although visual and auditory stimuli have distinct physical properties, another literature highlights the potential for redundant information across the modalities to facilitate cross-modal perception (e.g., Smith et al., 2016a, 2016b; Stevenage et al., 2017). Therefore, it would appear that person perception relies on both unimodal and potentially cross-modal information. However, little is known about the relative contribution of these sources of information, and how they might interact. In this study, we use a novel audio-visual sorting task that speaks directly to this question, requiring unimodal (face and voice) and cross-modal (face-voice) sorting. We ask whether such a paradigm might improve performance for both unimodal and cross-modal identity perception, with the availability of cross-modal person information facilitating stable representations (e.g., Burton et al., 2016).

### Unimodal identity perception

Unimodal visual and auditory information do not contribute equally to identity percepts. Whilst a voice is only audible when a person is speaking, a face can be viewed regardless of its owner’s actions (e.g., during sleep). Perhaps as a consequence, faces are found to be more reliable indicators of identity, such that voice processing accuracy is usually lower (see Barsics, 2014; Stevenage & Neil, 2014; Young et al., 2020). These differences in accuracy have in turn been attributed to vocal identity being encoded with less perceptual clarity or salience than facial identity, and being more subject to interference (Stevenage et al., 2011; Stevenage et al., 2013).

Nevertheless, there are also many examples of visual and auditory identity information being processed in similar ways, with effects such as averaging and distinctiveness being analogous in the two modalities (Barsics & Brédart, 2012; Bruckert et al., 2010; Langlois & Roggman, 1990). Thus, despite differences in accuracy, evidence for similarities between face and voice perception tend to dominate the literature. An example of these similarities is linked to the fact that faces and voices both exhibit notable within-person variability, with people looking and sounding very different across instances (Burton, 2013; Lavan, Burton, Scott, & McGettigan, 2019). The sources and nature of the variability may not be readily comparable across modalities (e.g., hairstyle or lighting for faces versus expressiveness or audience accommodation effects for voices), but the effect on perception is the same. Thus, while accuracy for unfamiliar face matching and unfamiliar voice matching can be relatively high, within-person variability nonetheless introduces errors (Bruce et al., 1999; Lavan et al., 2016; Smith et al., 2019). This is shown most clearly in identity sorting tasks, where participants are instructed to sort a set of naturally varying stimuli into different identities. In the identity sorting tasks, it is common to incorrectly perceive multiple images or recordings of the same unfamiliar person as representing a number of different people (Jenkins et al., 2011; Lavan, Burston, & Garrido, 2019; Stevenage et al., 2020). Johnson et al.’s (2020) results suggest that these similarities in findings across modalities may be underpinned by some common processes, as performance in face and voice sorting tasks was correlated, albeit weakly. Consistent with faces providing more reliable identity cues, Johnson et al. (2020) also found that face sorting was more accurate than voice sorting using a “free” identity sorting task, in which participants are unaware of the veridical number of identities (see also Jenkins et al., 2011; Lavan, Burston, & Garrido, 2019). Similarly, face advantages are also seen for “forced” sorting, where participants are informed how many identities are represented by the stimuli: In these tasks, accuracy is higher for both faces and voices compared with free sorting, but where forced face sorting tends to be near perfect (Andrews et al., 2015), voice sorting remains relatively error prone (Lavan, Merriman, Ladwa, et al., 2019).

### Cross-modal identity perception

When considering the many parallels between face and voice perception, the potential for integration and interaction across modalities is clear. In particular, evidence of cross-modal after-effects, cross-modal priming, and cross-modal associative priming provide compelling evidence that integration of unimodal cues occurs during identity perception (Bülthoff & Newell, 2017; Schweinberger et al., 2007; Stevenage et al., 2012; Stevenage et al., 2014; Zäske et al., 2010).

Redundant information across the two modalities likely plays a role in facilitating such cross-modal integration. Faces and voices provide a range of overlapping information, including cues to attractiveness, masculinity, femininity, and health (Collins & Missing, 2003; Saxton et al., 2006; Smith et al., 2016a). Several studies have consequently demonstrated that it is possible to match unfamiliar faces and voices across modality with low, but above chance, accuracy (Krauss et al., 2002; Mavica & Barenholtz, 2013; Smith et al., 2016a, 2016b; Stevenage et al., 2017). Overall, performance is more consistent when matching voices to dynamic faces compared with static faces (Kamachi et al., 2003; Smith et al., 2016b). However, notably there are also studies reporting chance performance for static and dynamic stimuli alike (Lavan, Smith, et al., 2020).

### The current study

In the current study, we present naturally varying face and voice stimuli in the same task and instruct participants to sort them into different identities, via a forced identity sorting paradigm (see Andrews et al., 2015; Lavan, Merriman, Ladwa, et al., 2019). Unlike the rigid experimental framework imposed by matching tasks, which may superficially restrict how auditory and visual identity information is processed, sorting tasks facilitate self-directed perception as listeners can freely choose which stimuli to attend to and have the opportunity to correct errors as they occur. At the same time, identity sorting tasks can readily capture and present participants with within-person variability at both the unimodal and cross-modal level. This may be of particular interest for the current study: Although within-person variability has mostly been discussed in the context of posing challenges to accurate identity perception (e.g., Jenkins et al., 2011; Lavan, Burston, & Garrido, 2019), recent work has highlighted the potential benefits of being exposed to within-person variability. This work suggests that within-person variability can facilitate the formation of robust unimodal identity representations (Burton, 2013; Burton et al., 2016; Lavan, Burton, Scott, & McGettigan, 2019). Face learning studies have indeed reported advantages for identity recognition after participants were trained with highly variable stimuli (as opposed to less variable stimuli; Murphy et al., 2015; Ritchie & Burton, 2017). This effect has been partially replicated for voice learning (Lavan, Knight, Hazan, & McGettigan, 2019). However, similar work looking at the effects of within-person variability for cross-modal identity judgements is largely missing. As such, it is possible that the stimuli used in a sorting task can potentially provide the building blocks for a stable, multimodal representation of an unfamiliar person (Burton et al., 2016). These audio-visual identity sorting tasks may therefore provide a novel way of observing how different sources of naturally varying identity information are dealt with in person perception, and how unimodal and cross-modal signals may be combined to inform and potentially improve identity perception accuracy.

Here, we first set out to observe self-directed identity sorting performance, comparing it with the more structured task of identity matching, which has been previously used to test both unimodal and cross-modal identity perception in the literature (Experiment 1). We then investigate the effect of processing strategy, by splitting the sorting task into unimodal and cross-modal stages (Experiment 2). Finally, we consider the effect of familiarity, testing whether minimal audio-visual training (1 minute of exposure) leads to improvements across sorting tasks (Experiment 3).

## Experiment 1: Comparing unimodal and cross-modal identity sorting to identity matching

In this experiment, we ran an initial identity sorting task, including naturally varying, dynamic face and voice stimuli. This experimental design enabled us to examine the overall accuracy for unimodal and cross-modal identity sorting when performed in conjunction.

We set out to compare accuracy for unimodal and cross-modal face and voice identity perception for this identity sorting task (Experiment 1A) to accuracy in identity matching tasks (Experiment 1B). As investigations of unimodal, and in particular cross-modal, identity perception have tended to adopt matching tasks rather than sorting tasks, this will enable us to contextualise our findings, facilitating comparisons with the previous literature. For identity sorting, participants are presented with a set of stimuli in an interactive drag-and-drop interface and are asked to sort the different stimuli into clusters, representing perceived identities. For identity matching, participants make iterative pairwise judgements about whether two stimuli (either two voice recordings, two videos of faces, or one voice recording and one video of a face) show the same person or two different people.

Based on the previous literature, we predicted that, across sorting and matching tasks, accuracy would be higher for unimodal face identity perception than for unimodal voice identity perception. We also expected that cross-modal face‐voice matching would elicit the lowest accuracy overall.

We did not have a directional prediction regarding differences in the accuracy for sorting and matching tasks: Matching tasks could lead to better performance as they force participants to make explicit pairwise judgements, while the self-directed nature of sorting tasks may lead to a less systematic assessment of the face and voice stimuli included in the task. On the other hand, in sorting tasks listeners are able to listen to recordings and view the videos again in a self-guided manner to potentially correct errors. This could in turn lead to higher accuracy for the sorting tasks.

## Methods

### Participants

Sixty participants were recruited for the identity sorting experiment (Experiment 1A). Out of these participants, 12 were excluded: 10 participants either failed our attention checks (see Materials) or did not follow instructions (see Procedure) and so created the wrong number of clusters. One participant was furthermore excluded because they sorted all voice recordings into one cluster and all the videos into another. A final participant was excluded because they recognised one of the identities included. Data from two participants was lost due to technical errors. The final sample thus included 46 participants (mean age = 28.5 years, *SD* = 6.1 years, 23 females). An independent sample of 51 participants (mean age = 27.5 years, *SD* = 6.6 years, 29 females) was recruited for the identity matching experiment (Experiment 1B). No participants were excluded from this sample. We intended to test around 50 participants per group, thus readily exceeding the sample sizes for most identity sorting studies (Jenkins et al., 2011; Lavan, Burston, & Garrido, 2019).

Both participant samples were recruited via the online recruitment platform Prolific.co. All participants were between the ages of 18 and 40 years, were native speakers of English, and were born in the United Kingdom and thus familiar with the accents used in our study. They had no reported hearing difficulties, normal or corrected-to-normal vision, and had a high approval rate on Prolific (>90%). Ethical approval was given by the local ethics committee (Project ID number: SHaPS-2019-CM-030). Participants were paid £2.25 for 20 minutes of participation for Experiment 1A and paid £3.75 for 30 minutes of participation for Experiment 1B.

### Materials

We created sets of face and voice stimuli from two Caucasian female British YouTubers with Standard Southern British English accents (Lara Jarvis and Kerry Whelpdale). Both are in their early 30s, vlogging about their lives as mothers with young children. From YouTube we gathered naturally varying stimuli for each modality (voice recordings, face videos): eight face videos and nine voice recordings of Kerry Whelpdale; nine face videos and eight voice recordings of Lara Jarvis. There were 34 stimuli in total, sampled from a 6-year period, including natural variability in terms of recording equipment and environment. All face and voice stimuli were extracted from different videos and scenes to minimise any incidental overlapping information (verbal content, background sounds). All stimuli featured full meaningful utterances of natural speech (e.g., “Did you notice that Stuart’s got rid of his beard a little bit?”) which spanned the full duration of the recording or video. Face videos and voice recordings were matched for duration, with both ranging from 1.9 seconds to 3.1 seconds (*M* = 2.5 seconds).

### Voice materials

The voice recordings featured no music, there was very minimal background noise, and no other voices were audible. The intensity of the recordings was root-mean-square normalised using Praat (Boersma & Weenink, 2019). The linguistic content was neutral, and nondiagnostic of identity in that it contained no personal information. All recordings were converted into MP3 files to reduce the overall file size.

### Face materials

In the videos the faces were broadly front-facing, with no occlusions (e.g., sunglasses); the full face was visible throughout. The videos were edited in Adobe Premiere Pro 2020. They were cropped to 300 × 300 pixels, showing from the top of the head to the collarbone. The videos did not include any sound.

### Vigilance trials

In addition to the materials described above, we included a vigilance task to check participants’ attention during the identity sorting task. For this purpose, a short video showing the face of Homer Simpson as well as an audio clip of Homer Simpson saying “I will be known as Homer J Simpson” were included. Homer Simpson was deemed to be a character that should be highly familiar to most participants, such that they would be able to match his face to his voice. If participants failed to sort the video of Homer’s face and audio recording of his voice into a cluster on their own, participants were excluded from the data set (see Participants).

## Procedure

### Identity sorting

All of the videos and voice recordings described above were added to the same PowerPoint slide including a plain white background. Each stimulus was represented by a numbered square (see Lavan, Burston, & Garrido, 2019; Lavan, Burston, Ladwa, et al., 2019; Lavan, Merriman, Ladwa, et al., 2019), and had a height of 2.29 and width of 2.29 cm on the slide. When the voice recording was played, the number was visible throughout. When the face video was played, the number was visible for 0.3 seconds before the video played. Once the video had finished, the numbered box appeared again. That is, the faces were only visible for the duration of the video. As in Johnson et al. (2020), participants were instructed not to change the size of the boxes or to pause the video (which would have allowed them to keep a face image on the screen).

**Fig. 1:**
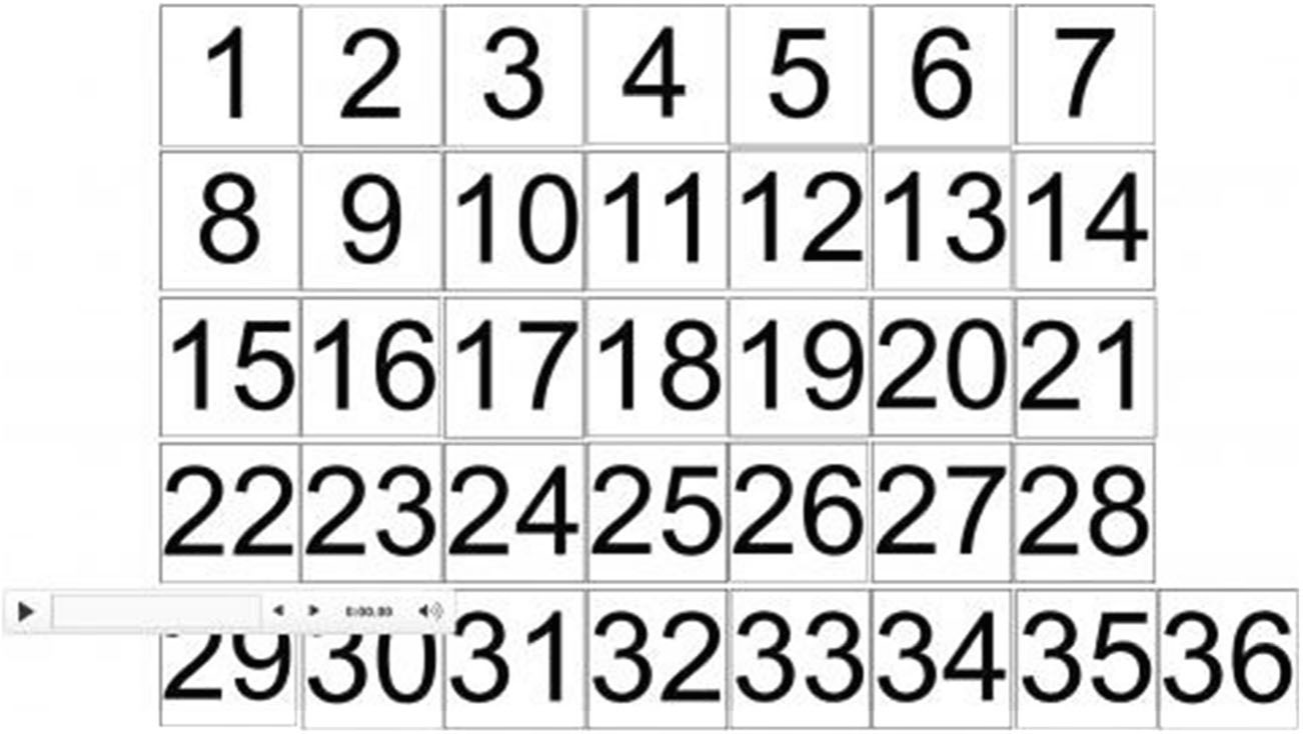
Illustration of the voice sorting task for Experiment 1A: Each numbered box represented a sound that could be played and replayed via a mouse click. Boxes were embedded on a PowerPoint slide and could thus be reorganised into separate clusters via drag-and-drop

The experiment was implemented on Qualtrics. After reading the information sheet and giving consent to take part in the study, participants received instructions about how to complete the task and then downloaded the PowerPoint slide including the 36 stimuli. The numbered boxes were arranged in a grid, ordered by number on the slide (see Fig. 1). This was a forced sorting task: Participants were told that three identities were present (that is, two females and the male third identity, Homer Simpson, acting as a vigilance trial), represented by both face and voice stimuli (e.g., Andrews et al., 2015; Lavan, Merriman, Ladwa, et al., 2019). The forced sorting task was used to optimise performance: “Free” identity sorting tasks, where participants are unaware how many identities are present, lead to systematic misperceptions (e.g., Jenkins et al., 2011; Lavan, Burston, & Garrido, 2019). Participants were able to play the voice recordings and face videos by clicking the numbered squares. They were instructed to sort the 36 stimuli by identity, by dragging and dropping the different stimuli into three (and only three) distinct clusters to represent the different perceived identities. They were told that two of these clusters needed to feature females, and one needed to feature a recognisable male character (vigilance trials).

Stimuli could be replayed as many times as participants felt necessary. Participants were aware that clusters did not have to be of similar size. The instructions furthermore highlighted that participants were required to combine faces and voices in the same clusters to complete the task correctly. Therefore, participants were sorting stimuli by identity both within modality (matching faces to faces, and voices to voices) and across modality (matching faces to voices). After completing the task, participants uploaded their sorted PowerPoint slide to a web server, from which the experimenters then retrieved the PowerPoint slide. Finally, participants were asked a number of debrief questions to assess whether they recognised any of the identities, and were asked to give free text responses regarding which strategy they used to solve the task (not formally analysed).

### Identity matching

All possible pairwise comparisons of the 34 stimuli were included in the experiment, excluding trials where the first and second stimulus were identical. In total there were 136 possible face-matching pairs, 136 possible voice-matching pairs, and 289 face‐voice-matching pairs. To avoid participant fatigue, pairs for each task (face matching, voice matching and face‐voice matching) were divided into four subsets of pairs of stimuli, with each subset being made up of a roughly equal number of same/different trials. In these subsets, each stimulus was repeated no more than four times for the single modality tasks, and no more than five times for the face‐voice matching task. For the face matching and voice matching tasks, the stimuli were randomly allocated to Position 1 or 2. In the face‐voice matching task, the order of stimuli was counterbalanced, with half of the trials featuring a face in Position 1, and half featuring a voice in Position 1. There were two possible orders (A or B) of each of the four subsets, with stimulus position reversed in Order B. Thus, in total there were eight versions of the experiment.

Visual catch trials were used in face matching blocks, in which the text instruction “please select ‘same person’” was shown. Auditory catch trials were used in voice-matching blocks, in which the instruction “same” or “different” was given in a synthetic male voice, created via the Speech Synthesis Manager of the Mac OS. Both types of catch trial were used in face‐voice-matching blocks.

The experiment was implemented on the Gorilla Experiment Builder (www.gorilla.sc; Anwyl-Irvine et al., 2020). After reading the information sheet and giving consent to take part in the study, participants were required to pass a headphone check (Woods et al., 2017). They were then randomly allocated to one of the eight versions of the experiment.

Each participant completed three separate counterbalanced blocks of face matching, voice matching, and face‐voice matching. The order of trials within blocks was fully randomised. Participants were told that they would only see two different women throughout the experiment. In the face‐voice-matching condition, they were informed that the face in the video and the voice in the recording were not saying the same thing, to prevent participants from using speech reading to reach a decision (Kamachi et al., 2003).

The two stimuli were presented sequentially in each trial. The interstimulus interval was 700 ms, during which a central fixation point was visible. Following the presentation of the stimulus in Position 2, two boxes appeared side by side, “same person” on the left and “different people” on the right. Participants clicked one of the boxes to register their response, and were then prompted to click “continue” to progress to the next trial. They were not able to revisit trials or view stimuli more than once.

Catch trials were randomly inserted throughout the blocks to ensure that participants were paying attention. There were four catch trials in the face and voice matching blocks, and eight catch trials in the face‐voice matching block.

## Data analyses

For each participant completing the identity sorting task, PowerPoint slides were coded for pairwise accuracy: We created a list of all possible pairwise combinations of the stimuli within and across modalities (unimodal [face, voice] and cross-modal [face‐voice]). A pair of stimuli from the same identity was coded as 1 if sorted into the same cluster (i.e., accurately “told together”) or 0 if sorted into different clusters. The reverse was the case for cells representing a pair of stimuli from different identities, such that ‘1’ represented a correct response (i.e., listeners accurately “told apart” these two stimuli), and ‘0’ represented an incorrect response (see also Lavan, Burston, & Garrido, 2019). Vigilance trials were excluded from all analyses.

These pairwise combinations also apply to the stimuli presented in the identity matching tasks: Unimodal face sorting performance is reflected in pairs comprising two videos, unimodal voice sorting is reflected in pairs comprising two audio recordings, and cross-modal face‐voice sorting is reflected in pairs comprising a video and an audio recording. As in the sorting task, there were “same identity” and “different identity” pairs for each of these three modality combinations.

To assess how the type of task affects accuracy in our experiments, we analysed the binary accuracy data using generalised linear mixed models (GLMMs) implemented in the lme4 package (Bates et al., 2014) in the R environment. Significance of the main effects and interactions was established via log-likelihood tests by dropping effects of interest from the appropriate model. For example, to establish whether the three-way interaction is significant, we dropped this three-way interaction from the model including all effects. To test for the significance of the two-way interactions, we dropped the relevant two-way interaction from the model that included all three two-way interactions.

## Results

**Fig. 2:**
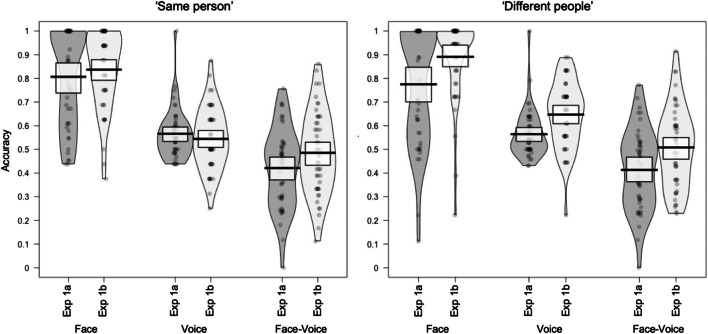
Mean accuracy per participant plotted for Experiment 1A (sorting) and Experiment 1B (matching) by modality and trial type. Boxes indicate 95% confidence intervals

The accuracy for Experiments 1A and 1B is plotted for each modality (face, voice, and face‐voice) per trial type (same person, different people) in Fig. 2.

We first assessed whether accuracy for the different trial types and modalities was different from chance for both experiments. For this purpose, we ran a GLMM with Experiment (matching/sorting), Trial Type (same/different), and Modality (face/voice/face‐voice) entered as fixed effects in the model with no intercept. All interactions were included. Each stimulus in a pair was included as a separate random effect. In total there were three random effects: Stimulus 1, Stimulus 2, and participant.

We obtained 95% confidence intervals by simulating the posterior distributions of the cell means in R (arm package, Version 1.6; Gelman & Su, 2013). If CIs do not include 50%, accuracy in the respective condition is different from chance. In our study, accuracy was above chance for both experiments and trial types for faces (all 95% CIs [ >75.0%, >80.0%]) and for voices (all 95% CIs [>50.6%, >59.4%]). For cross-modal face‐voice matching, accuracy was at chance (all 95% CIs [>44.9%, >51.9%]) but was below chance for cross-modal face‐voice sorting (all 95% CIs [<39.1%, <45.1%]).

We ran a further GLMM to assess how accuracy was affected by the experimental task. In this intercept model, Experiment, Trial Type, and Modality were again entered as fixed effects in the model. All interactions were included. Participant, Stimulus 1, and Stimulus 2 of each of the stimulus pairs were entered as random effects.

There was a significant three-way interaction between Experiment, Trial Type, and Modality, χ^2^(2) = 12.51, *p* = .002, as well as significant two-way interactions between Trial Type and Experiment, χ^2^(1) = 23.14, *p* < .001, and Experiment and Modality, χ^2^(2) = 17.48, *p* < .001. The two-way interaction between Trial Type and Modality was not significant, *χ*^*2*^(2) = 5.27, *p* = .072. See Table 1 for the model outputs.

**Table 1:** Coefficients and standard errors (reported on a log-odds scale) for the full model including the three-way interaction for the data from Experiment 1A (sorting) and Experiment 1B (matching)^a^

Predictors	Log-odds	Standard error
(Intercept)	1.43	0.08
Main effect of trial type		
Trial type (same)	0.19	0.06
Main effect of experiment		
Experiment (1B)	0.24	0.13
Main effect of modality		
Modality (face‐voice)	−1.76	0.06
Modality (voice)	−1.13	0.07
Two-way interaction Trial Type × Experiment		
Trial Type (Same) × Experiment (1B)	0.67	0.16
Two-way interaction Trial Type × Modality		
Trial Type (Same) × Modality (Face‐voice)	0.16	0.07
Trial Type (Same) × Modality (Voice)	0.19	0.08
Two-way interaction Experiment × Modality		
Experiment (1B) × Modality (Face‐voice)	0.02	0.12
Experiment (1B) × Modality (Voice)	−0.34	0.14
Three-way interaction Trial Type × Experiment × Modality		
Trial Type (Same) × Experiment (1B) × Modality (Face‐voice)	−0.55	0.17
Trial Type (Same) × Experiment (1B) × Modality (Voice)	−0.24	0.19

^a^ The reference categories are “different identity” judgements for trial type, Experiment 1A for experiment, and unimodal face judgements for modality

To follow up the three-way interaction, we ran six post hoc tests to compare accuracy split by Trial Type and Modality for Experiments 1A and 1B. These post hoc tests were implemented using the R package emmeans (Version 1.4; Lenth, 2019). This enabled us to further examine how the choice of tasks (sorting vs. matching) across experiments interacts with accuracy in each modality. The post hoc tests showed that accuracy was higher for Experiment 1B compared with Experiment 1A for all modalities (face, voice, face‐voice) in “different identity” judgements (all βs < −.32, all *SE*s > .09, all *z*s < 2.99, all *p*s < .003). For “same identity” judgements, a similar pattern emerged for cross-modal face‐voice identity judgements (β = −.26, *SE* = .09, *p* = .005) and faces, although this effect was not significant (β = −.24, *SE* = .13, *p* = .072). For “same identity” voice judgements accuracy was numerically (but not significantly) lower for matching compared with sorting (β = −.10, *SE* = .11, *p* = .353).

## Discussion

In Experiments 1A and 1B, we aimed to establish a baseline level of accuracy for unimodal and cross-modal identity sorting, and further link this level of accuracy in the sorting task with the accuracy found via more established matching tasks.

Overall, accuracy was higher for identity matching than for identity sorting, although this effect was modulated both by type of trial and the stimulus modalities: For example, the accuracy advantage for identity matching (vs. sorting) was larger for “different identity” judgements compared with “same identity” judgements. Further, where “same identity” judgements were more accurate for face and face‐voice identity matching compared with sorting, the opposite numerical pattern was seen for voice identity. The difference across tasks was, however, relatively subtle, with mean performance across tasks never varying by more than 6% in the “same identity” judgements or 11% for the “different identity” judgements in any modality. This is surprising, as the two tasks differ substantially in terms of stimulus presentation (sorting: all stimuli for both modalities are encountered within the same interface; matching: stimuli are presented by modality and in pairs), how participants are required to interact with the stimuli (sorting: self-initiated and self-selected stimulus presentation, responses given within an unconstrained drag-and-drop interface; matching: fixed, pairwise stimulus presentation, two-way forced-choice responses) and in the specific judgements that are required (sorting: grouping stimuli by identity; matching: same/different identity judgements). Given these substantial differences, we might have predicted that performance would have been strikingly divergent.

Independent of the task, unimodal and cross-modal identity perception followed the predicted pattern: Accuracy was highest for faces, substantially lower for voices, and lowest for face‐voice identity perception. Despite supporting our predictions, aspects of the results were surprising: For example, Andrews et al. (2015) report virtually error-free performance for a restricted identity sorting task with unfamiliar faces, in which participants were made aware of the veridical number of identities included in the task. In our experiment, accuracy was high, but errors in both “same identity” judgements (“telling people together”) and “different identity” judgements (“telling people apart”) are still apparent. This difference can be attributed to our design choice of making the dynamic videos of the faces disappear after video playback to better match face sorting to voice sorting (see also Johnson et al., 2020). For Andrews et al. (2015), the images of faces were visible throughout the sorting task, reducing the working memory load.

Similarly, accuracy for voice identity sorting was somewhat lower than previously reported for a restricted voice identity sorting task (Lavan, Merriman, Ladwa, et al., 2019). A possible explanation for these differences may be that the two voices used in the current study were selected to be of a similar voice quality, of a similar age, and speaking with the same accent. The two voices used on Lavan, Merriman, Ladwa, et al. (2019) were sampled opportunistically from the TV show *Breaking Bad* (Hank Schrader and Walter White), such that their age and accents were likely less well-matched, potentially leading to better accuracy. For voice identity matching, accuracy was also relatively low: These findings echo previous voice matching studies that include within-person variability (i.e., different categories of speaking style/nonverbal vocalisation; Lavan et al., 2016; Smith et al., 2019), further highlighting the difficulties that such within-person variability can pose to accurate identity perception.

Finally, accuracy for face‐voice matching was close to 50%, and thus at chance. This may not be surprising, given the generally low, albeit above-chance accuracy, for dynamic face‐voice matching tasks reported in the literature (Kamachi et al., 2003; Lander et al., 2007; Mavica & Barenholtz, 2013; Smith et al., 2016a, 2016b; Stevenage et al., 2017 but see Lavan, Smith, et al., 2020, for chance-level dynamic face‐voice matching). Intriguingly, accuracy for face‐voice *sorting* was below 50%, suggesting that the inclusion of multiple variable instances of the faces and voices of our two identities did not result in more accurate cross-modal identity perception. Indeed, the results suggest that participants may systematically match the wrong faces to the voice and vice versa.

Overall, accuracy for cross-modal identity perception may be somewhat lower in our experiment than is usually reported in the literature. There are several possible explanations for this: In contrast to the stimuli typically used in face‐voice matching tasks, our task included multiple visual and auditory stimuli representing the same identity, thus sampling natural within-person variability. Having immediate access to multiple variable stimuli representing the same identity may have aided cross-modal identity sorting (Burton et al., 2016; Lavan, Burton, Scott, & McGettigan, 2019). At the same time, the stimulus set and task had the potential to be detrimental to face‐voice matching, given the challenges within-person variability can pose to unfamiliar identity perception (Jenkins et al., 2011; Lavan, Burston, & Garrido, 2019). Furthermore, identity-specific effects have frequently been reported for face‐voice matching, where the faces and voices for some identities can more accurately be matched than for others (e.g., Smith et al., 2016b; Stevenage et al., 2017). The current experiment may have sampled a pair of identities for which face‐voice matching is particularly difficult. Given partially conflicting findings regarding above-chance versus chance-level face‐voice matching performance in the existing literature, and the fact that cross-modal identity perception accuracy is significantly affected by task (sorting vs. matching), we stress that the overall levels of matching accuracy observed here should not be overinterpreted.

## Experiment 2: Separating unimodal and cross-modal identity sorting

From Experiment 1A it is unclear how participants used unimodal and cross-modal information to complete the sorting task. Specifically, because both types of sorting (unimodal and cross-modal) were happening within a single task, we are unable to determine whether their strategy in one type of sorting might have affected performance in the other. For example, if participants had been able to access shared cues to identity across faces and voices, asking them to integrate cross-modal information during identity sorting may have strengthened the identity representations and therefore supported unimodal identity sorting. Alternatively, if the shared cues to identity across modalities are unreliable, being asked to integrate information across modalities may have hindered unimodal identity sorting. 

In an attempt to address this question and separate out the unimodal and cross-modal task elements, we adapted our sorting paradigm for a new sample of participants who were required to complete the task in separate stages. Specifically, they first completed unimodal identity sorting tasks, with cross-modal identity judgements required only *after* this stage had been completed. If cross-modal information supports sorting, accuracy for the unimodal face and voice sorting should be lower in Experiment 2 compared with Experiment 1A, where unimodal and cross-modal sorting occurred at the same time. However, if having to integrate cross-modal information hindered unimodal identity sorting, accuracy for the unimodal face and voice sorting should be higher in Experiment 2 compared with Experiment 1A.

## Methods

### Participants

Sixty-two participants between the ages of 18 and 40 years were recruited via the online recruitment platform Prolific.co using the same criteria and payment as for Experiment 1A. 14 participants were excluded: 12 participants either failed our attention checks (see Materials) or created the wrong number of clusters, thus rendering their data unusable. These data were never analysed (see Procedure). Two further participants were excluded because they recognised one of the identities included. The final sample thus included 48 participants (mean age = 27.5 years, *SD* = 6.7 years, 30 females).

### Materials

The materials used were the same as those described for Experiment 1A.

For the current experiment, however, the catch trials for the sorting task were Stuart Jarvis, Lara Jarvis’ husband. As an unfamiliar male, Stuart was chosen in case seeing and hearing Homer made participants in this experiment guess that there was a relationship between the faces and voices.

### Procedure

**Fig. 3:**
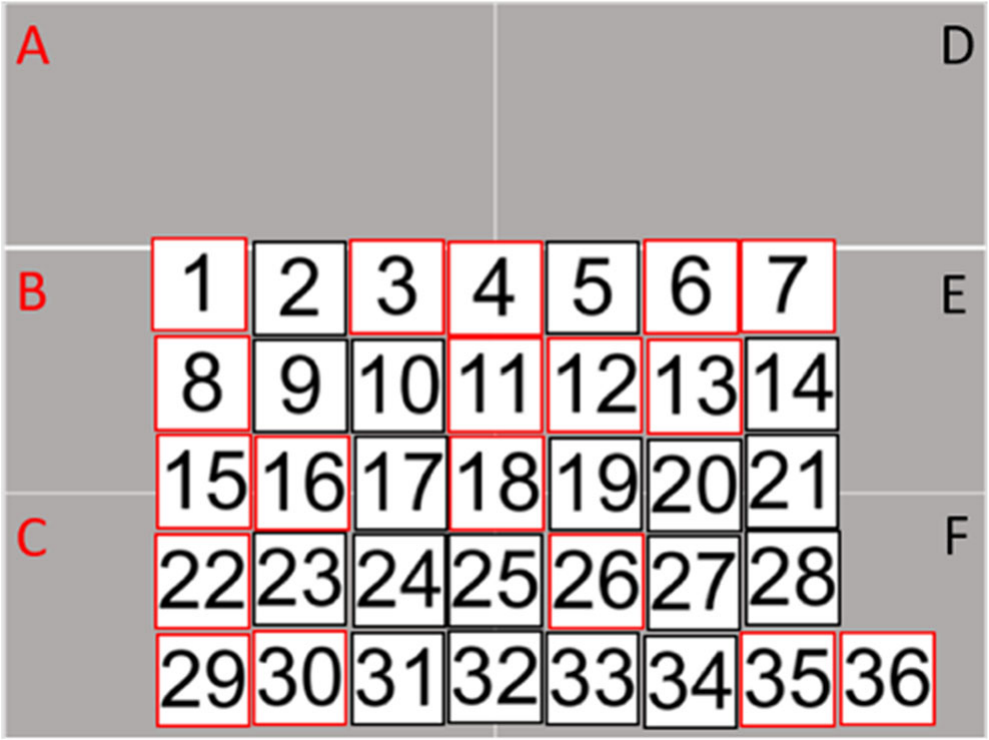
Illustration of the materials for the voice sorting task for Experiment 2

Each stimulus was represented by a numbered square box. Boxes with a red outline indicated voices, and boxes with a black outline indicated faces. To guide participants’ sorting behaviour, the PowerPoint slide featured a grey background separated into six rectangles (two × three configuration). The rectangles provided a labelled area for each of the clusters (red for voices; black for faces, see Fig. 3) so that the labels could be used to indicate which faces and voices belonged together in the second stage of the experiment.

Participants were instructed to sort the stimuli into six different identity clusters: three clusters for voices in the A, B, and C rectangles, and three clusters for faces in the D, E, and F rectangles. They were told that two clusters per modality would need to feature females, one would need to feature a man. Participants were therefore required to sort the modalities independently of one another: Similar to Experiment 1A, they had to do unimodal sorting (matching faces to faces and voices to voices), but unlike in Experiment 1A, no cross-modal sorting (matching faces to voices) was necessary. It was only after participants had uploaded their completed PowerPoint slide that they were informed that the three voices and three faces actually came from the same three identities (i.e., for every face cluster there was a corresponding voice cluster, and vice versa). Participants then completed a face‐voice matching task, where they indicated which of the face clusters and voice clusters they had compiled belonged to each other. Specifically, participants were asked to look back at their sorted slide and indicate how the identities in the ‘voice’ clusters (A, B, C) match the ‘face’ clusters (D, E, F). There was no time limit on this task and participants were thus able to revise their answer as many times as they felt necessary. This post hoc cluster-level sorting across modalities thus conceptually replicated the procedure for sorting the different items by identity on the PowerPoint slide. In the post-test questionnaire, some participants indicated that they suspected during the unimodal sorting task that the face and voice stimuli belonged to the same identity. Accuracy for these participants did, however, not differ from the accuracy of listeners who reported no such suspicion, so all 48 participants were retained in the analysis.

## Data analysis

For this experiment, we directly compared participants’ accuracy to the data reported for the sorting task from Experiment 1A above. Data were processed in the same way as described in Experiment 1: All data was coded in terms of pairwise accuracy. The information from the cross-modal sorting (matching face and voice clusters by identity) was taken into account by merging the stimuli in the face and voice clusters that each participant had indicated as belonging to the same identity in a cross-modal cluster. The data from Experiment 2 are thus in the same format as the data in Experiment 1, making the two experiments directly comparable. Data were then analysed in the same way as described for Experiment 1.

## Results

**Fig. 4:**
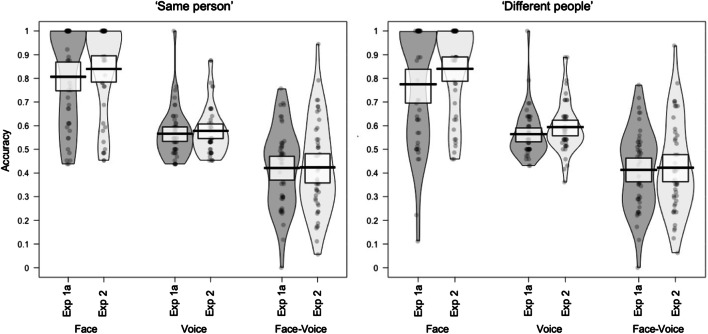
Mean accuracy per participant plotted for Experiment 1A and Experiment 2 by modality and trial type. Boxes indicate 95% confidence intervals

Accuracy for Experiments 1A and 2 is plotted for each modality (face, voice and face‐voice) per trial type (same person, different people) in Fig. 4. As for the previous experiments, accuracy for Experiment 2 was above chance for face sorting (all 95% CIs [>79.9%, >84.7%]) and for voice sorting (all 95% CIs [>55.5%, >63.1%]), but was below chance for cross-modal face‐voice sorting (all 95% CIs [<40.8%, <48.1%]).

We ran a further GLMM to assess how accuracy was affected by our experimental manipulations. This GLMM included Experiment, Trial Type, and Modality as fixed effects and participant and stimulus as random effects. Neither the three-way interaction between Experiment, Trial Type, and Modality, χ^2^(2) = 2.61, *p* = .272, nor the two-way interactions between Trial Type and Experiment, χ^2^(2) = 5.19, *p* = .075, Experiment and Modality, χ^2^(1) = 3.25, *p* = .071, or Trial Type and Modality, χ^2^(2) = 5.17, *p* = .075, were significant. However, there was a significant two-way interaction between Experiment and Modality, χ^2^(2) = 42.49, *p* < .001. There was also no main effect of Trial Type, χ^2^(1) = .82, *p* = .364. Please see Table 2 for model outputs.

**Table 2:** Coefficients and standard errors (reported on a log-odds scale) for the full model including the three-way interaction comparing accuracy for Experiment 1A and Experiment 2^a^

Predictors	Log-odds	Standard error
(Intercept)	1.47	0.09
Main effect of trial type		
Trial type (same)	0.19	0.06
Main effect of experiment		
Experiment (2)	0.27	0.11
Main effect of modality		
Modality (face‐voice)	−1.79	0.06
Modality (voice)	−1.18	0.07
Two-way interaction Trial Type × Experiment		
Trial Type (Same) × Experiment (2)	−0.2	0.09
Two-way interaction Trial Type × Modality		
Trial Type (Same) × Modality (Face‐voice)	−0.16	0.07
Trial Type (Same) × Modality (Voice)	−0.19	0.08
Two-way interaction Experiment × Modality		
Experiment (2) × Modality (Face‐voice)	−0.25	0.08
Experiment (2) × Modality (Voice)	−0.2	0.09
Three-way interaction Trial Type × Experiment × Modality		
Trial Type (Same) × Experiment (2) × Modality (Face‐voice)	0.17	0.11
Trial Type (Same) × Experiment (2) × Modality (Voice)	0.12	0.12

^a^ Reference categories for trial type are the “different” judgements, for experiment is Experiment 1A and for modality are face judgements

Post hoc tests to follow up the two-way interaction between Experiment and Modality implemented in emmeans (Version 1.4; Lenth, 2019) revealed that while accuracy for face sorting increased between Experiment 1A and Experiment 2 ( β = −.38, *SE* = .10, *p* < .001), this was not the case for voice sorting, where only a small numerical improvement was apparent (β = −.11, *SE* = .10, *p* = .252) or face‐voice sorting, where there was no improvement ( β = −.03, *SE* = .09, *p* = .749).

## Discussion

First, the experiment shows that accuracy increases significantly for unimodal face sorting and numerically for voice sorting when participants are not required to integrate identity information across modalities. One possible explanation for this is that being required to integrate identity information across modalities has the potential to be disruptive to accurate unimodal face sorting, and is unhelpful to voice sorting. Second, accuracy for cross-modal (i.e., face‐voice) sorting remained the same compared with Experiment 1A. Indeed, accuracy was still below 50%, indicating that the perceptual decisions to systematically match the wrong face with the wrong voice observed for identity sorting in Experiment 1A persisted in Experiment 2.

As in the comparison of Experiments 1A and 1B, the changes in accuracy between Experiments 1A and 2 are, however, small. Our experimental manipulation, which separated unimodal from cross-modal sorting therefore had no major effect on sorting accuracy. It is unclear whether this is due to listeners having largely employed similar strategies across Experiment 1A and Experiment 2, or whether the current task manipulation truly only has minor effects of accuracy.

## Experiment 3: Exploring the effect of minimal training on unimodal and cross-modal identity sorting

Performance in our face‐voice identity sorting tasks (Experiments 1A and 2) was low, and participants tended to perceive the wrong faces and voices as belonging together, resulting in below-average accuracy. In Experiment 3, we therefore examined how minimal training (and thus minimal familiarity) with the identities affects unimodal and cross-modal identity sorting. We predicted that minimal familiarity should overall increase accuracy for both unimodal and cross-modal sorting. However, we expected the biggest benefits to occur for cross-modal face‐voice sorting: Through our minimal training, participants were explicitly shown which faces and voices go together, thus providing them with essential information to support accurate cross-modal matching. We expected that this training would lead to an increase in accuracy for the cross-modal element of the identity sorting task in particular.

## Methods

### Participants

Fifty participants between the ages of 18 and 40 years were recruited via the online recruitment platform Prolific.co with the same recruitment criteria as in Experiments 1A and 2. One participant was excluded because they recognised one of the identities, and another person was excluded because they formed one cluster with only voice recordings and another cluster with only face videos in them. The final sample included 48 participants (mean age = 26.0 years, *SD* = 6.5 years, 35 females).

### Materials

For the minimal training, we extracted a 58-second extract from a video on Laura Jarvis’ YouTube channel, featuring her and Kerry Whelpdale taking turns to describe the contents of their handbags. The video was filmed in January 2018. No other people feature in it, both women’s faces are fully visible throughout, and each of them speak for roughly equal periods of time. The video was edited in Adobe Premiere Pro, measured 540 × 960 pixels, and was shown in .mp4 format. The sorting task was identical to the one in Experiment 1A.

### Procedure

Apart from the following exceptions, the materials and methods were identical to Experiment 1A. Participants were informed that they were going to watch a short, 1-minute video of two women talking to each other. They were instructed to watch the video once carefully, paying particular attention to the women’s faces and voices. They were told that during the main part of the experiment they would be asked to make some judgements based on the faces and voices of these two women.

Participants were unable to proceed to the next screen until they had watched the video in full. They were then asked three simple questions to ensure that they had been paying attention during the video (for example, “Did the women discuss lipsticks and lip gloss?”; correct answer: “Yes”). Having answered these questions correctly, participants progressed to the main part of the experiment, where they received the sorting instructions. From this point on, the procedure was identical to that of Experiment 1A.

## Data analysis

As in the previous experiment, we directly compared participants’ accuracy for Experiment 3 to the accuracy reported in Experiment 1A. Data were analysed in the same way as described in the previous experiments.

## Results

**Fig. 5:**
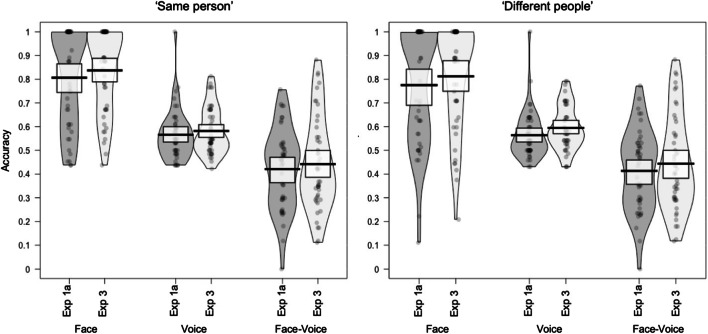
Mean accuracy per participant plotted for Experiment 1A and 3 by modality and trial type. Boxes indicate 95% confidence intervals

Accuracy for Experiments 1A and 3 is plotted for each modality (face, voice and face‐voice) per trial type (same person, different people) in Fig. 5. Accuracy for Experiment 3 was above chance for both “same identity” and “different identity” judgements for faces (all 95% CIs [>78.2%, >83.1%]) and for voices (all 95% CIs [>55.7%, >63.1%]), but was below chance for cross-modal face‐voice sorting (95% CIs [all <39.9%, all <46.8%]).

We ran a further GLMM to assess how accuracy was affected by our experimental manipulations. This GLMM included Experiment, Trial Type and Modality as fixed effects and participant and stimulus as random effects. There was no significant three-way interaction between Experiment, Trial Type, and Modality, χ^2^(2) = .19, *p* = .907. There were, however, significant two-way interactions between Trial Type and Modality, χ^2^(2) = 14.56, *p* = .001, and Modality and Experiment, χ^2^(2) = 9.86, *p* = .007. The two-way interaction between Experiment and Trial Type was not significant, χ^2^(1) = 1.27, *p* = .260. Please see Table 3 for model outputs.

**Table 3:** Coefficients and standard errors (reported on a log-odds scale) for the full model including the three-way interaction comparing accuracy for Experiment 1A and Experiment 3^a^

Predictors	Log-odds	Standard error
(Intercept)	1.46	0.09
Main effect of trial type		
Trial type (same)	−0.19	0.06
Main effect of experiment		
Experiment (3)	0.27	0.12
Main effect of modality		
Modality (face‐voice)	−1.17	0.07
Modality (voice)	−1.78	0.06
Two-way interaction Trial Type × Experiment		
Trial Type (Same) × Experiment (3)	0.01	0.09
Two-way interaction Trial Type × Modality		
Trial Type (Same) × Modality (Face‐voice)	0.19	0.08
Trial Type (Same) × Modality (Voice)	0.16	0.07
Two-way interaction Experiment × Modality		
Experiment (3) × Modality (Face‐voice)	−0.18	0.09
Experiment (3) × Modality (Voice)	−0.18	0.08
Three-way interaction Trial Type × Experiment × Modality		
Trial Type (Same) × Experiment (3) × Modality (Face‐voice)	−0.25	0.1
Trial Type (Same) × Experiment (3) × Modality (Voice)	0.03	0.1

^a^ Reference categories for trial type are the “different identity” judgements, for experiment is Experiment 1A and for modality are face judgements

To follow up the two-way interaction between Modality and Experiment, we again ran post hoc tests implemented using the R package emmeans (Version 1.4; Lenth 2019) to examine how the minimal training affected accuracy. None of the pairwise comparisons of the accuracy for Experiment 1A and Experiment 3 by Trial Type or Task was significant after correcting for six multiple comparisons (alpha = .008; βs range from −.09 to −.28, *SE*s > .10, *p*s > .012).

## Discussion

Minimal training somewhat improved accuracy for unimodal and cross-modal sorting. However, this effect resulted in only small numerical improvements in accuracy that were not statistically significant: This is perhaps surprising, especially for performance on cross-modal identity sorting, since participants were shown how the faces and voices match. Why did this short training then not lead to substantial improvements of participants’ performance for unimodal face and voice sorting? The exposure may have been too brief, or was perhaps not varied enough to facilitate the building of stable identity representations that would enable listeners to better generalise across the within-person variability included in the stimuli.

We had predicted that cross-modal face‐voice sorting performance would benefit most from minimal training, since participants were given, albeit briefly, the information necessary to successfully integrate cross-modal identity information. Nonetheless, the patterns in the data, suggesting that participants systematically match the wrong faces and voices to each other, have not been completely removed: Cross-modal face‐voice sorting accuracy remained below 50% for both “same" identity and “different” identity judgements.

Thus, although small improvements in accuracy were apparent, the minimal training we implemented (~1 minute of audio-visual exposure to two identities) does not appear to allow participants to gather meaningful information about the faces and voices sufficiently to support significantly higher accuracy for unimodal or cross-modal identity sorting.

## General discussion

In this study, we examined unimodal and cross-modal identity perception using a sorting paradigm with naturally varying stimuli. Specifically, we asked how unimodal and cross-modal identity perception may interact in this experimental setup. In Experiment 1, we observed performance in our novel audio-visual sorting task, and compared accuracy with an identity matching task: Such matching tasks are more frequently used in the person perception literature, particularly in the context of cross-modal identity. Accuracy was generally higher for the matching task than the sorting task, although the differences in accuracy were at times relatively subtle. We therefore conclude that there is only a modest effect of experimental task on the accuracy of unimodal and cross-modal identity perception. In Experiment 2, we showed that separating unimodal and cross-modal identity sorting increased accuracy for unimodal sorting—an effect that was only significant for faces—while the accuracy of cross-modal sorting remained the same. This may suggest that using cross-modal information has the potential to be detrimental to unimodal identity sorting. Experiment 3 showed that minimal audio-visual exposure to the identities improved overall sorting accuracy numerically, although these improvements were not significant. Crucially, no major improvement was apparent for cross-modal identity perception, suggesting that substantially longer and more varied exposure is necessary to link face and voice identity information in a unified multimodal representation of a person.

Across all experiments, we replicate previous findings from the literature, showing that face identity perception is generally more accurate than voice identity perception (e.g., Barsics, 2014). Similarly, accuracy for cross-modal identity perception was low (e.g., Kamachi et al., 2003; Lander et al., 2007; Smith et al., 2016a, 2016b). In fact, in our sorting experiments, below-chance accuracy was apparent for cross-modal identity perception. Specifically, faces belonging to one person tended to be sorted into the same identity as voices belonging to the *other* person. Overall, these observations fit with existing findings suggesting that some identities are perceived to have better matching faces and voices than others (Huestegge, 2019; Mavica & Barenholtz, 2013;Smith et al., 2016b ; Stevenage et al., 2017). However, we note again that we refrain from inferring too much from the below-chance accuracy. Accuracy for cross-modal identity perception was not below chance in the identity matching task for Experiment 1B, suggesting that (cross-modal) identity perception judgements are at least partially task dependent. Furthermore, if unimodal judgements tend to be prioritised over cross-modal judgements in a sorting task, one incorrect face‐voice decision in a sorting task might implicate numerous individual stimuli within an identity “cluster” (cf. matching tasks, in which such below-chance performance could more likely reflect systematic inaccuracy across multiple same-different judgements). This would certainly have been the case in Experiment 2, where a single cross-modal decision was taken after the unimodal sorting had been completed.

What can our experiments tell us about the proposed integration and interaction of auditory and visual information during unimodal and cross-modal identity judgements? Previous work on identity perception and learning using naturally varying face stimuli has proposed that exposure to variability enables participants to build stable unimodal representations (Burton et al., 2016; see also Andrews et al., 2015; Murphy et al., 2015). In our experiments, the low accuracy for cross-modal identity judgements suggests that participants were not able to use unimodal within-person variability to identify shared information across modalities. On the other hand, we predicted that having access to cross-modal information during sorting tasks may have aided unimodal identity perception. However, having to attend to cross-modal information impeded accurate unimodal identity perception overall, an effect that was significant for faces and numerical only for voices: We therefore observed an increase in accuracy for unimodal sorting in the absence of cross-modal sorting (Experiment 2). Taken together, our findings therefore suggest that in the context of identity sorting tasks, listeners failed to successfully use cross-modal information to inform unimodal identity judgements, and vice versa.

Identity sorting tasks have a number of features that differ from matching tasks, enabling us to observe how unimodal and cross-modal information interact. Participants are able to perceive identity in a largely self-directed manner: All stimuli are available to be viewed or played at any point, participants can freely select which stimuli to view or listen to, perception strategies can be chosen and adapted, and errors can be corrected. We therefore argue that this task should provide an ideal environment to integrate identity-related cues both within and across modalities. From this perspective, it is therefore all the more surprising that cross-modal and unimodal information were not found to be mutually informative.

We already speculated that listeners may not have used the within-person variability to build robust multimodal representations of the faces and voices in the study. We further speculate that this variability may have actually reduced the informativeness of the cross-modal information. Previous studies have shown that there are concordant cross-modal cues to, for example, attractiveness, masculinity, femininity, and health in people’s faces and voices when rated in the absence of within-person variability (e.g., Smith et al., 2016a). Thus, attractiveness and other physical or trait-related percepts can in principle be informative for cross-modal identity judgements: Attractive voices tend to go with attractive faces. However, recently studies in trait perception reported that the perceived attractiveness, trustworthiness, and dominance of facial images and voice recordings of the same person can vary substantially in the presence of within-person variability (Lavan, Mileva, et al., 2020; Todorov & Porter, 2014). If participants attempted to use cues such as attractiveness or health to inform their decisions, the within-person variability included in our stimuli may have destabilised the identity percepts, rendering cross-modal cues less diagnostic and thus disrupting cross-modal identity perception.

Previous face‐voice matching studies (e.g., Krauss et al., 2002; Mavica & Barenholtz, 2013; Smith et al., 2016a, 2016b; Stevenage et al., 2017) have sampled between-person variability, presenting several identities across multiple trials. However, these studies have not sampled within-person variability: Participants make matching decisions based on only one voice recording or one face image/video featuring each identity. As in previous unimodal sorting studies (e.g., Johnson et al., 2020; Lavan, Merriman, Ladwa, et al., 2019) we include only two identities in order to sample within-person variability and address whether this might support cross-modal perception. We required a high number of stimuli so that we could sample across a long time period (6 years), as well as across different recording equipment and environments. Including additional identities would have weakened our design, making it necessary to reduce the number of stimuli that could be presented per identity to make the task manageable. We accept that identity-specific effects might have operated here, and that alternative identities might have been easier to group (Smith et al., 2016b; Stevenage et al., 2017). However, the decision to include only two identities does not undermine our conclusions about the potentially destabilising effect of within-person variability information in cross-modal perception (Experiment 1) or the unreliable nature of cross-modal identity information (Experiments 1 and 2), even following minimal familiarity (Experiment 3).

The inability to make accurate cross-modal identity judgements is intriguing in the context of person perception in naturalistic settings: During the process of familiarisation, auditory and visual identity information become linked to form a multimodal representation of a person (von Kriegstein et al., 2005; von Kriegstein et al., 2006). However, the current findings emphasise that cross-modal information pertaining to a person’s identity—or perceptual access to this information—appears to be either unreliable or cannot be used efficiently. Our findings thus put into focus that although shared information may be present (e.g., health, attractiveness, etc.) under certain circumstances, the two modalities appear to be largely independent sources of identity information. Due to the relative independence of individual modalities, facial and vocal information cannot be readily integrated during identity perception, either when experienced in isolation (i.e., unimodally), or even after minimal cross-modal exposure (see Experiment 3). It is to date unclear how multimodal representations are built, and how relatively independent visual and auditory information are integrated into a unified percept. Future research therefore needs to determine how much and what kind of exposure (i.e., unimodal or multimodal) is necessary to successfully match (familiarised) faces and voices.
